# Lnc90386 Sponges miR-33-5p to Mediate *Mycoplasma gallisepticum-*Induced Inflammation and Apoptosis in Chickens *via* the JNK Pathway

**DOI:** 10.3389/fimmu.2022.887602

**Published:** 2022-06-27

**Authors:** Yingfei Sun, Yingjie Wang, Mengyun Zou, Tengfei Wang, Lulu Wang, Xiuli Peng

**Affiliations:** Key Laboratory of Agricultural Animal Genetics, Breeding and Reproduction, Ministry of Education, Huazhong Agricultural University, Wuhan, China

**Keywords:** *Mycoplasma gallisepticum* (MG), miR-33-5p, JNK signaling pathway, Lnc90386, ceRNA

## Abstract

*Mycoplasma gallisepticum* (MG) is one of the most important pathogens, that causes chronic respiratory disease (CRD) in chickens. Long non-coding RNAs (lncRNAs) are emerging as new regulators for many diseases and some lncRNAs can function as competing endogenous RNAs (ceRNAs) to regulate mRNAs by competitively binding to miRNAs. Here, we found that miR-33-5p was significantly up-regulated both in MG-infected chicken embryonic lungs and chicken embryo fibroblast cells (DF-1), and Lnc90386 negatively correlated with miR-33-5p. miR-33-5p, as a new regulator for MG infection, repressed apoptosis, inflammatory factors in DF-1 cells by targeting JNK1. Further analyses showed that Lnc90386 sponged miR-33-5p to weaken its inhibitory effect on JNK1, forming the ceRNA regulatory network. Furthermore, knockdown of Lnc90386 significantly inhibited apoptosis and inflammatory factors, and promoted DF-1 cells proliferation. However, co-treatment with miR-33-5p inhibitor and Lnc90386 siRNA showed that knockdown of Lnc90386 could partially eliminate the inhibiting effect of miR-33-5p inhibitor on inflammation, cell apoptosis and proliferation. In conclusion, Lnc90386 sponges miR-33-5p to defend against MG infection by inhibiting the JNK signaling pathway.

## Introduction

*Mycoplasma gallisepticum* (MG) infection causes chronic respiratory disease (CRD), which is a major infectious disease that endangers the development of the poultry industry worldwide (1–3). Many studies have shown that MG adheres to the epithelial cells of the respiratory tract through its adhesion protein (pMGA), and then crosses the cell membrane through microtubules. Upon these processes, MG can induce inflammation damage and apoptosis by releasing genetic material and enzymes into the host cell (4, 5). MG can exist for a long time and be difficult to be eliminated from infected birds, leading to serious economic losses in the poultry industry worldwide (6–8). Therefore, it is urgent to find a new and effective way to block the transmission of MG or treat MG-induced inflammatory damage and apoptosis (9–11). MG-HS, a virulent strain, is used in this study. pMGA1.2, the crucial adhesion protein on the surface of MG-HS, is responsible for MG-HS to propagate in animal tissues through binding to apolipoprotein A–I (ApoA–I) on the host (12).

miRNAs are a group of abundant non-coding small RNAs found in plants and animals. They can repress the expression of target genes by binding to their 3’UTR sequences through seed sequences (13). Growing evidence has demonstrated that miRNAs play a significant role in avian diseases. For example, miRNAs can regulate avian influenza, Marek’s disease, infectious bursal disease and avian mycoplasmosis (14). In addition, our previous studies have also highlighted the critical role of miRNAs (such as miR-181a-5p, miR-223 and miR-24) during MG infection (13, 15, 16). miR-33-5p belongs to the miR-33 family and is highly conserved among species (17, 18). Several studies reveal that miR-33 is differentially expressed in a variety of poultry diseases, such as avian influenza (19), and Newcastle disease (20), suggesting that miR-33 may play an important role in various pathogenic microorganism infections. Our previous miRNAs deep sequencing showed that miR-33-5p was significantly up-regulated in MG-HS infection, indicating its potentially significant role in MG-HS infection (21).

C-jun N-terminal kinase 1 (JNK1) is a member of the mitogen-activated protein kinase (MAPK) family which is widely expressed in various tissue cells. The JNK signaling pathway centered on JNK1 can be activated by a variety of factors, and plays a crucial role in cell apoptosis and immune response. In addition, this pathway is closely related to the occurrence and development of various respiratory diseases. Numerous studies have shown that JNK1 is involved in the regulation of lung diseases, inflammation and apoptosis (22). According to these reports, JNK1 is inhibited by viral NS1 protein thus blocking apoptosis of infected cells (23). JNK1-deficient mice can decrease influenza A burden in viral pneumonia, yet display worsened morbidity (24). Furthermore, peptidoglycan from Staphylococcus aureus requires JNK1 to drive IL-8 production in lung type II cells (25). However, the role of JNK1 in MG-induced CRD remains unclear.

Long non-coding RNAs (lncRNAs), a type of ncRNAs longer than 200nt, have been demonstrated to be involved in the regulation of lung disease progression (26). For example, lncRNA HOTTIP inhibits the expression of miR-774-5p and thus promotes lung tissue fibrosis (27). lncRNA FEZF1-AS1, as an oncogenic regulator, binds to miR-516b-5p to promote proliferation and invasion of lung cancer cells (28). And the silencing of lncRNA MEG3 enhances the binding between miR-7b and NLRP3 to ameliorate LPS-induced acute lung injury (29). These findings suggest that lncRNAs, as sponges for miRNAs, play important roles in a variety of biological processes (lung diseases). However, it is unclear whether miR-33-5p mediates MG infection and whether lncRNAs could interact with miR-33-5p to influence the expression of miR-33-5p target genes and thus regulate the infection process of MG.

In this study, DF-1 cells spread throughout the body were used as an *in vitro* model to investigate the mechanism of operation of Lnc90386-miR-33-5p during the infection of MG. We identified that miR-33-5p inhibits JNK signaling pathway *via* targeting JNK1 to repress MG-induced inflammation and apoptosis, and Lnc90386 as a ceRNA to alleviate the effect of miR-33-5p on negatively regulating JNK1 in MG infection. Our results reveal a novel MG-induced CRD regulatory model that is composed of miR-33-5p, Lnc90386 and JNK1, and explore their ceRNA system in MG-induced inflammation and apoptosis.

## Materials and Methods

### Mycoplasma Strains and Infection Experiments

MG-HS, a virulent strain, is reported in detail in our previous studies (30). The concentration of MG-HS in this study was 10^10^CCU/mL. DF-1 cells and CP-II cells were cultured in a 6-wells plate and treated MG. After 24h infection, we isolated the total RNA or protein from infected cells. In addition, White Leghorn specific-pathogen-free (SPF) chicken embryos were used for MG-HS infection experiments. The experimental method was detailed in our previous reports (31). In short, the chick embryo allantoic cavity was injected with 300 ul of MG on 9-day of incubation. Then, samples of chicken embryonic lung tissue were collected at 6, 7, 8, and 9 days post-infection (15, 16, 17 and 18 days after hatching).

### Construction of Plasmid and Dual-Luciferase Reporter Assay

The wild-type and mutant 3’UTR DNA fragments of JNK1 covering the predicted binding sites of miR-33-5p were successfully replicated. The psiCHECK™-2-JNK1-3’UTR (wild type and mutant) vector was constructed by combining the luciferase vector psi-CHECK™-2 (Promega, Madison, WI, USA) with JNK1-3’UTR (wild type and mutant). Similarly, we cloned the CDS of JNK1 or Lnc90386 into the pcDNA3.1 vector. The primers are described in **Table 1**.

**Table 1:** Sequences of DNA primers.

Name	Primer Sequence (5′-3′)
	**Primers for CDS Cloning**
**JNK1-CDS-F**	CAAGCTGGCTAGCGTTTAAACTTAAGCTTATGAGCAGAAGCAAGCGTGAC
**JNK1-CDS-R**	GGTTTAAACGGGCCCTCTAGACTCGAGTCATTGCTGCACCTGTGCTA
**Lnc90386-CDS-F**	GGCTAGCGTTTAAACTTAAGCTTGGTACCAAGAAAACCTATGATCGTGCAATTA
**Lnc90386-CDS-R**	CTGGACTAGTGGATCCGAGCTCGGTACCACATCAATATATTTTTATTTTTATAAATTATTCC
**Mut-Lnc90386-CDS-F**	TATAATCACGATAAACTTGTAATTGAAGAACAGTCT
**Mut-Lnc90386-CDS-R**	TACAAGTTTATCGTGATTATATATATCTTCTAAGCTATCTCCTT
	**Primers for Dual-Luciferase Reporter**
**JNK1 3’UTR-F**	TTCCTCGAGTTCGAAGTTTTATGCTTGGTC
**JNK1 3’UTR-R**	AATGCGGCCGCCTAGACGTCCTGGATACACC
**Mut-JNK1 3’UTR-F**	GAATAATACATTACAGCATAAATAAACTGTTTACTTCTAGCTTG
**Mut-JNK1 3’UTR-R**	GTAAACAGTTTATTTATGCTGTAATGTATTATTCATGAAGTATCCTC
**Luc-Lnc90386-F**	AGCAGTAATTCTAGGCGATCGCTCGAGGAAAACCTATGATCGTGCAATTAAG
**Luc-Lnc90386-R**	GTTTAAACGAATTCCCGGGCTCGAGTATACCTACAACTGGTGCTTTCAGC
**Mut-Luc-Lnc90386-F**	TATAATCACGATAAACTTGTAATTGAAGAACAGTCT
**Mut-Luc-Lnc90386-R**	TACAAGTTTATCGTGATTATATATATCTTCTAAGCTATCTCCTT
	**Primers for RT-qPCR**
**RT-gga-miR-5S**	AACTGGTGTCGTGGAGTCGGC
**gga-5s-rRNA-F**	CCATACCACCCTGGAAACGC
**gga-5s-rRNA-R**	TACTAACCGAGCCCGACCCT
**RT-gga-miR-33**	CTCAACTGGTGTCGTGGAGTCGGCAATTCAGTTGAGGCAATGCA
**gga-miR-33-F**	GGGTGCATTGTAGTTGCATTGC
**gga-miR-33-R**	ACTGGTGTCGTGGAGTCGGC
**IL-1β-F**	CTACACCCGCTCACAGTCCT
**IL-1β-R**	TCGGTGCCGCTCATCACACA
**TNF-α-F**	GGACAGCCTATGCCAACAAG
**TNF-α-R**	ACACGACAGCCAAGTCAACG
**GAPDH-F**	CCTCTCTGGCAAAGTCCAAG
**GAPDH-R**	TTGATGTTGCTGGGGTCACG
**JNK1-F**	CAATAGGATCGGGAGCGCAA
**JNK1-R**	TCGTTCATGGTCTAGCTCCA
**JUN-F**	CTCAGCAACTTCAACCCCAA
**JUN-R**	CTTTGATTCTCTCCTGCGAC
**FOS-F**	GACAGCCTCACCTACTACCC
**FOS-R**	AAATCCTGCGAGTTGACGGG
**FAS-F**	CTGCTCCTCGTCATTGTGTT
**FAS-R**	CTCCACAGGTAATTTCTCGC
**BCL-2-F**	AAGCAAGCGTGACAAC
**BCL-2-R**	ATCATAGGCTGCACATAC
**CASP8-F**	GGGTGTCTCCGTTCAGGTAT
**CASP8-R**	CATCTCTCCTTCACCAAGTAAGT
**CASP9-F**	TGCCGACGTTCCATAGAGTC
**CASP9-R**	ACGATGTCTGACACCCGAAG
**Lnc90386-F**	CTTGATTCTGCAAGGCGCTC
**Lnc90386-R**	TCCACCTCCCAGTCAAGTCA
**Lnc56449-F**	TTACAGAGCTCAGCTGCACC
**Lnc56449-R**	GCACGCATCGAAATTCCCTC
**Lnc66351-F**	CTCCTCTCCCTCTGGCTTCT
**Lnc66351-R**	ATTTGCAAAGAACACCGGGC
**Lnc68634-F**	CCACAGCTGCCTGAACTACA
**Lnc68634-R**	GACTGACCTGCCCAGTTTGA
**Lnc71825-F**	CGGCGCACACAAATTTAGCT
**Lnc71825-R**	GGCATTTGTAGGCCCTCACT
**Lnc81972-F**	TGCAGAAAGGCTTGCCAAAC
**Lnc81972-R**	AGCCCAGCTCCTTCTAGGAA
**Lnc86360-F**	GTTTGCACGTTTCCCACCTC
**Lnc86360-R**	CCCACGGGAAGCTTTCAGAT
**Lnc88478-F**	CCCACTGCCTCCTAACGTTT
**Lnc88478-R**	GGTTGACTTCGCGAGTTTCG

Dual-luciferase reporter assay is detailed in our previous reports (16). In a nutshell, when cells reached 80-90% confluence, using Lipofectamine 2000 (Invitrogen Life Technologies, USA) each co-transfected cells with wild-type or mutant reporter plasmid (200 ng) and 10 pmol of the indicated RNA oligonucleotides. Then, the luciferase activity in each group was detected by using an automatic microplate reader (Bio-Rad, Hercules, CA, USA) in accordance with the dual luciferase reporter gene detection kit instructions (Promega, Madison, WI, USA) according to the manufacturer’s protocol.

### DNA Primers and RNA Oligonucleotides

In this experiment, total sequences of DNA primers were adopted, which are presented in **Table 1**. In addition, miR-33-5p mimics (denoted as miR-33-5p) are double-stranded RNAs synthesized to simulate naturally occurring mature miR-33-5p, whereas the inhibitor (denoted as miR-33-5p-Inh) are chemically modified antisense single-stranded RNAs that silence the endogenous miRNAs by sequence complementarity. A random miRNA mimics that had not been found to suppress any chicken target genes (denoted as miR-33-5p-NC), and a random miRNA inhibitor that had not been found to promote any chicken target genes (denoted as miR-33-5p-Inh-NC) were also designed and synthesized to serve as the negative controls. All RNA oligonucleotides were designed and synthesized by GenePharm (Shanghai, China). The RNA oligonucleotides sequences are shown in **Table 2**.

**Table 2:** Sequences of RNA oligonucleotides.

Name	Sequences (5′–3′)
**miR-33-5p mimics**	GUGCAUUGUAGUUGCAUUGC
	AAUGCAACUACAAUGCACUU
**miR-33-5p NC**	UUCUCCGAACGUGUCACGUTT
	ACGUGACACGUUCGGAGAATT
**miR-33-5p inhibitor**	GCAAUGCAACUACAAUGCAC
**miR-33-5p inhibitor-NC**	CAGUACUUUUGUGUAGUACAA
**Si-Lnc90386**	GGGCUUAAGCGUUCAGCUATT
	UAGCUGAACGCUUAAGCCCTT
**siRNA NC**	UUCUCCGAACGUGUCACGUTT
	ACGUGACACGUUCGGAGAATT

### Overexpression or Inhibition of miR-33-5p, Lnc90386 and JNK1

Once 80-90% confluence was achieved, each group of cells was transfected with mimics, inhibitor, plasmids and siRNA respectively. At 48h after transfection, TRNzol (TIANGEN, Beijing, China) was used to harvest total cells.

### RNA Isolation and Quantitative Real-Time PCR

According to the manufacturer’s instructions, total RNA was isolated from post-infected and non-infected cells *via* TRNzol Universal Reagent kit (TIANGEN, Beijing, China). Then, RNA was inverse transcribed to cDNA with the first strand cDNA synthesis kit (Cat No.11119-11141; Yeasen, Shanghai, China) and reverse transcription PCR (RT-PCR).

### Cell Proliferation and Apoptosis Assays

The Cell Counting Kit-8 (CCK-8, DOJINDO, Shanghai, China) was used for cell proliferation experiments. Cells were inoculated on a 96-well plate at 2x10^4^ cells per well. Each group of cells was separately transfected with different oligonucleotides or plasmids using Lipofectamine™3000 and each group had 6 biological replicates. Next, MG-HS (7µL, 10^10^ CCU/mL) was utilized to infect DF-1 cells for 2 hours. At 12h, 24h, and 36h post-transfection, the cell proliferation curve was measured by the CCK-8 kit according to the manufacturer’s instructions. Transfection treatments were described above. Annexin V, FITC apoptosis detection kit (DOJINDO) was used to test the cell apoptosis. Each group was repeated three times.

### ELISA

The grouping of transfection treatments was described above. Forty-eight hours after transfection, the supernatants were collected and the pro-inflammatory cytokines (IL-1β and TNF-α) levels were detected with enzyme-linked immunosorbent assay kits (Bio Legend, San Diego, CA) according to the manufacturer’s directions.

### Western Blot

Forty-eight hours after transfection, DF-1 cells were lysed in RIPA buffer (Beyotime, Nantong, China) supplemented with 100 mM phenyl methane sulfonyl fluoride (PMSF) to exact total protein. Protein concentrations were measured by the Pierce BCA Protein Assay Kit (Transgen, Shanghai, China). An equal amount of protein was separated by 12% SDS-polyacrylamide gel electrophoresis (Beyotime, China) and blocked with 5% skim milk for 1h. Then, the membranes were separately probed with p-JUN (ABclonal, AP0048), p-FOS (ABclonal, AP0038), p-JNK1 (ABclonal, AP0631), Bcl-2 (ABclonal, A19693), Caspase8 (ABclonal, A0215), Caspase9 (ABclonal, A18676), Caspase3(ABclonal, A19654), GAPDH (Abmart, M20024) overnight at 4°C with a final dilution of 1:5000 (v/v). Finally, the membrane was incubated with the secondary antibody for 1h after TBST washing. The enhanced chemiluminescence (ECL) detection system (Bio-Rad) was used to detect protein expression.

### Statistical Analysis

Data were presented as the mean ± SD. Student’s t-test was used to determine significant differences between groups. A value of *p* < 0.05 was considered statistically significant and *p* < 0.01 considered extremely significant. (*p < 0.05, **p < 0.01).

## Results

### miR-33-5p Is Remarkably Increased in MG Infection

**Figure 1:**
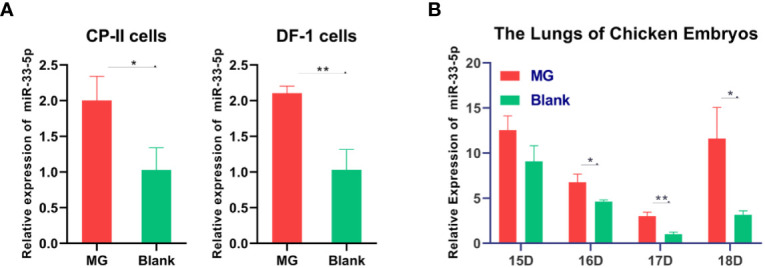
Relative miR-33-5p expression level in MG-infected cells and chicken embryonic lung tissue. **(A)** The relative level of miR-33-5p in MG-infected DF-1 cells and CP-II cells. Cells cultivated in 6-well culture dishes were treated with MG. After 24h treatment, total RNA of infected cells were extracted using TRNzol Universal. The level of miR-33-5p-infected cells was detected by qPCR; **(B)** The relative level of miR-33-5p in MG-infected chicken embryo lungs. Total RNA were extracted from infected chicken embryo lungs on days 6-9 post-infection (equivalent to days 15-18 of eggs hatching) using TRNzol Universal. Then, the level of miR-33-5p in MG infected embryo chicken lungs was determined through qPCR. Above data was corrected *via* 5s-RNA as the internal quantitative control gene. All measurements shown were the mean ± SD from three independent experiments, each with three replicates. (*p < 0.05, **p < 0.01).

Previous studies found that miR-33-5p was significantly up-regulated in MG infection by deep sequencing (21). The qPCR result showed that miR-33-5p was remarkably up-regulated both in DF-1 cells and CP-II cells with MG infection compared with the control group (**Figure 1A**). In addition, we observed that miR-33-5p expression was significantly higher in the lungs of the MG-infected chicken embryos than in the non-infected group on the 7th–9th days of post-infection (equivalent to the 16th–18th days of egg hatching) (**Figure 1B**).

### miR-33-5p Affects pMGA1.2 Expression

**Figure 2:**
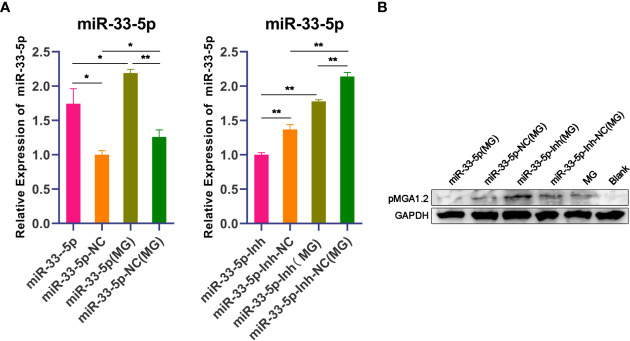
miR-33-5p inhibits pMGA1.2 expression in DF-1 cells. DF-1 cells were transfected with miR-33-5p, miR-33-5p-NC, miR-33-5p-Inh or miR-33-5p-Inh-NC and were incubated for 24h and then infected MG for 24h. **(A)** qPCR was used to detect the expression of miR-33-5p, 5s-RNA was used as the internal control; **(B)** Western blot was used to detect the adhesion protein pMGA1.2 of MG. GAPDH was used as the internal control. (*p < 0.05, **p < 0.01).

pMGA1.2, the major adhesin protein of MG-HS, is required for MG-HS infection (12). To further evaluate the effect of miR-33-5p on the MG infection in DF-1 cells, miR-33-5p, miR-33-5p-NC, miR-33-5p-Inh or miR-33-5p-Inh-NC were transfected into DF-1 cells. Then, the DF-1 cells were treated with 200 µL of MG at 10^10^ CCU/mL for 24h. qPCR and Western blot were used to respectively determine the expression levels of miR-33-5p and pMGA1.2. As expected, miR-33-5p mimics had further up-regulated the expression of miR-33-5p induced by MG infection, whereas miR-33-5p inhibitor diminished its expression (**Figure 2A**). Furthermore, we found that overexpression of miR-33-5p led to a down-regulation of pMGA1.2 protein expression in DF-1 cells, while the opposite result was obtained when miR-33-5p was suppressed (**Figure 2B**).

### miR-33-5p Inhibits MG-Induced Inflammation and Apoptosis

**Figure 3:**
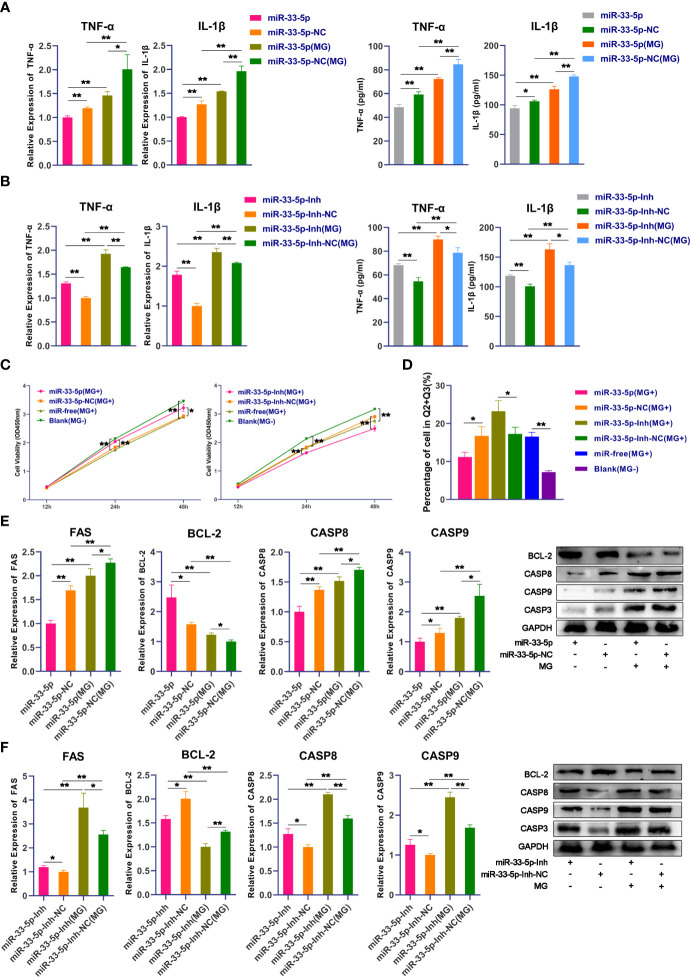
miR-33-5p inhibits MG infection-induced inflammation and apoptosis. DF-1 cells were transfected with the indicated RNA oligonucleotides and infected with MG. **(A)** The expression levels of pro-inflammatory cytokines (TNF-α and IL-1β) after treatment with miR-33-5p mimics were detected by qPCR and Elisa; **(B)** The expression levels of pro-inflammatory cytokines (TNF-α and IL-1β) after treatment with miR-33-5p inhibitor were detected by qPCR and Elisa; **(C)** CCK-8 kit was used to detect cell proliferation at 12, 24, 48h post-infection; **(D)** The cells were stained with Annexin V- PI and analyzed by flow cytometer after 24h post-infection; **(E)** The expression of apoptosis marker genes after treatment with miR-33-5p mimics was detected by Western blot and qPCR; **(F)** The expression of apoptosis marker genes treatment with miR-33-5p inhibitor was detected by Western blot and qPCR. GAPDH was used as the internal control. Data was expressed as the mean ± SD of three independent experiments, each with three replicates. (*p < 0.05, **p < 0.01).

Next, we further explored the biological functions of miR-33-5p on MG infection. qPCR and ELISA results showed that the expressions of pro-inflammatory cytokines IL-1*β* and TNF-*α* were significantly down-regulated in the miR-33-5p mimics group compared with the miR-33-5p NC group (**Figure 3A**). Conversely, when the expression of endogenous miR-33-5p was inhibited, the release of pro-inflammatory cytokines was significantly increased (**Figure 3B**). In the MG-infected group, transfection with miR-33-5p mimic significantly down-regulated the expressions of MG-induced IL-1*β* and TNF-*α*, but transfection with miR-33-5p inhibitor showed the opposite results (**Figures 3A, B**).

In addition, we examined the effect of miR-33-5p on cell proliferation by using the CCK-8 kit. Data showed that MG infection significantly inhibited DF-1 cell proliferation. Overexpression of miR-33-5p alleviated the inhibition of MG-induced cell proliferation, whereas suppression of miR-33-5p exacerbated the loss of MG-induced cell proliferation (**Figure 3C**). Furthermore, cell apoptosis assay showed that upon MG infection, overexpression of miR-33-5p resulted in a reduction in apoptosis, while inhibition of miR-33-5p led to an opposite result (**Figure 3D**). To further explore how miR-33-5p regulates MG-induced cell apoptosis, the expression of apoptosis-related genes (including pro-apoptotic and anti-apoptotic genes) were examined by qPCR and Western blot. We found that pro-apoptotic genes including FAS, Casp3, Casp8 and Casp9 were significantly increased, while the anti-apoptotic gene BCL-2 was significantly suppressed after MG infection. Overexpression of miR-33-5p inhibited the expression of MG-induced FAS, Casp3, Casp8 and Casp9, but up-regulated the BCL-2 expression (**Figure 3E**). However, in the miR-33-5p inhibitor group, the opposite trend was observed (**Figure 3F**).

### JNK1 Is a Direct Target of miR-33-5p

**Figure 4:**
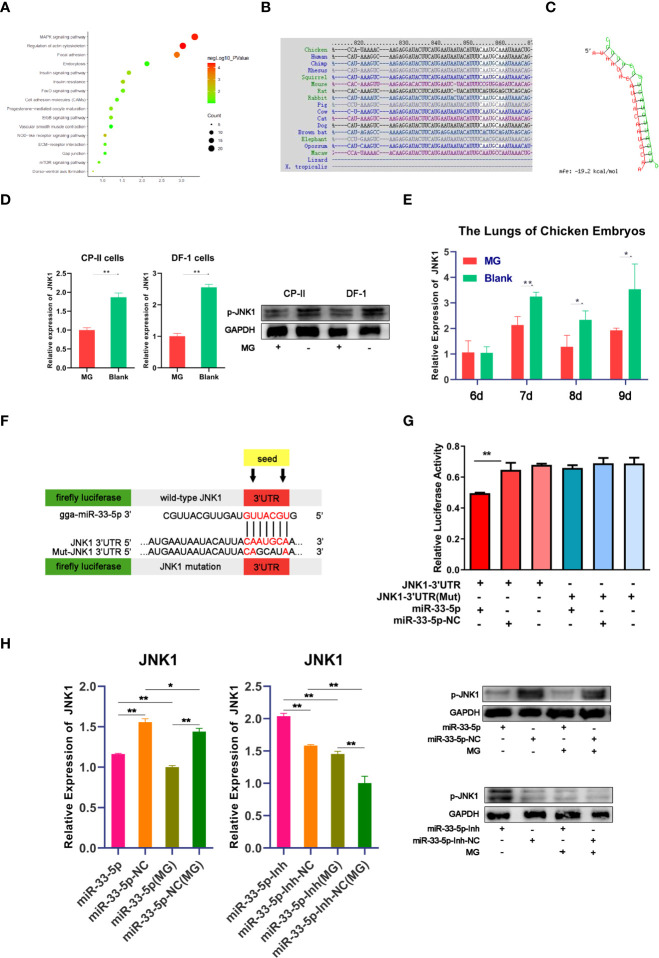
miR-33-5p negatively regulates JNK1 in DF-1 cells. **(A)** The KEGG enrichment result of target genes of miR-33-5p; **(B)** Alignment of JNK1 3′UTR in different species; **(C)** The secondary structure of the RNA duplex of miR-33-5p and the JNK1 3’UTR target site; **(D)** The JNK1 expression was detected by qPCR and Western blot in MG-infected DF-1 cells and CP-II cells, GAPDH was used as the internal control; **(E)** The relative expression level of JNK1 in chicken embryo lungs from chicken embryo lungs on days 6-9 post-infection (equivalent to days 15-18 of eggs hatching) using qPCR, GAPDH was used as the internal control; **(F)** psiCHECK-2 dual-luciferase reporter vector containing the 3′UTR (wild-type or mutant) of JNK1; **(G)** DF-1 cells were co-transfected with Luc-JNK1 3′UTR (wild-type or mutant) and miR-33-5p mimics. At 24h post-transfection, the cells were assayed for both firefly and renilla activity through a dual-luciferase glow assay; **(H)** The mRNA and protein expression of JNK1 after treatment with miR-33-5p mimic/miR-33-5p inhibitor, GAPDH was used as the internal control. (*p < 0.05, **p < 0.01).

It is well known that miRNAs mediate complex biological processes by partially binding to the 3’UTR of target genes (32). In order to explore the function of miR-33-5p in MG infection, potential target genes were predicted by TargetScan (http://www.targetscan.org) and miRDB (http://www.mirdb.org/miRDB/index.html). Analysis of the KEGG database identified that the target genes of miR-33-5p were mainly enriched in the MAPK pathway (**Figure 4A**). The prediction result of TargetScan showed that the target site’s sequence in the 3’UTR of JNK1 is highly conserved across species (**Figure 4B**). The minimum free energy (MFE) of the RNA duplex was -19.2 kcal/mol (**Figure 4C**) by RNAhybrid, which suggested a powerful combination between miR-33-5p and JNK1. Meanwhile, we also found that JNK1 expression was down-regulated in both MG-infected cells and chicken embryo lungs, which was exactly opposite to the expression pattern of miR-33-5p (**Figures 4D, E**). To further confirm the targeting relationship between miR-33-5p and JNK1, the dual-luciferase reporter vectors containing either the wild-type or the mutant 3’UTR of JNK1 were constructed (**Figure 4F**). Compared with the control group, miR-33-5p mimics could significantly reduce the luciferase activity of the reporter vector containing wild-type 3’UTR, but had no obvious effect on the luciferase activity of mutant 3’UTR reporter vector (**Figure 4G**). These results indicated that miR-33-5p could be complementarily bound to 3’UTR of JNK1. In addition, the results of qPCR and Western blot showed that miR-33-5p mimics significantly inhibited JNK1 expression in both MG-infected and uninfected DF-1 cells, while miR-33-5p inhibitor markedly up-regulated JNK1 expression compared with the control group (**Figure 4H**).

### miR-33-5p Inhibits the JNK Pathway by Targeting JNK1

**Figure 5:**
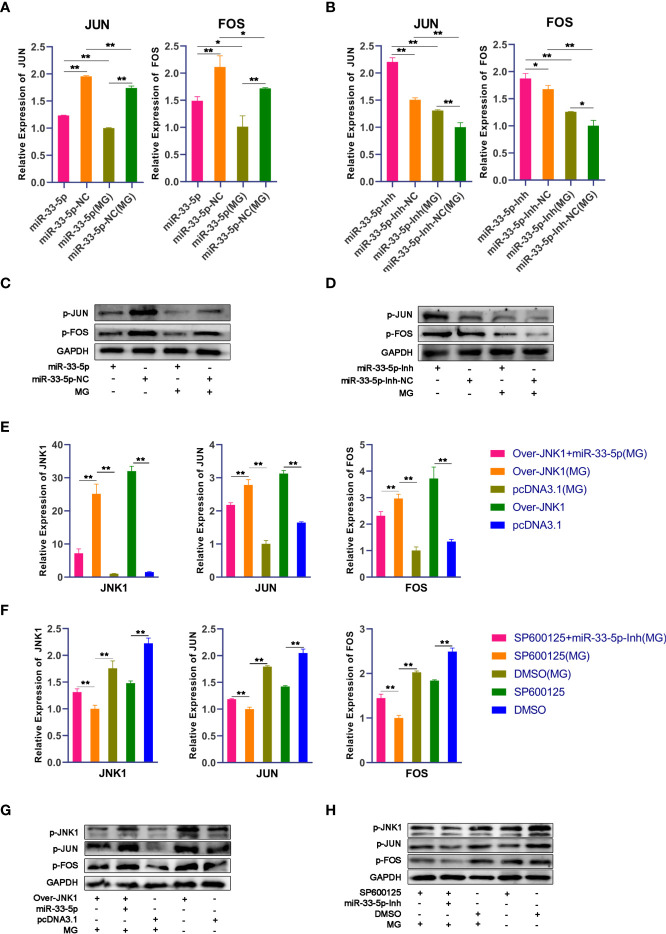
miR-33-5p suppresses the JNK pathway by directly inhibiting the expression of JNK1. DF-1 cells were transfected with the indicated RNA oligonucleotides and infected with MG. JNK1, JUN and FOS mRNA levels and protein levels were measured by qPCR and Western blotting, respectively. **(A)** The relative mRNA expression level of JNU and FOS after treatment with miR-33-5p mimics; **(B)** The relative mRNA expression level of JNU and FOS after treatment with miR-33-5p inhibitor; **(C)** The protein expression of JNU and FOS after treatment with miR-33-5p mimics; **(D)** The protein expression of JNU and FOS after treatment with miR-33-5p inhibitor; **(E)** The relative mRNA expression level of JNK1, JUN and FOS after co-transfection of overexpression JNK1 vector and miR-33-5p mimics; **(F)** The relative mRNA expression level of JNK1, JUN and FOS after co-transformation of SP600125 and miR-33-5p inhibitor; **(G)** The protein expression of JNK1, JUN and FOS after co-transfection of overexpression JNK1 vector and miR-33-5p mimics; **(H)** The protein expression of JNK1, JUN and FOS after co-transformation of SP600125 and miR-33-5p inhibitor. GAPDH was used as the internal control. (*p < 0.05, **p < 0.01).

Next, we further studied the functional mechanism of miR-33-5p in MG infection. It has been well reported that the JNK signaling pathway is the core pathway through which JNK1 exerts a regulatory role (33, 34). The results of qPCR and Western blot showed that the mRNA and phosphorylated protein expressions of JUN and FOS in the MG infection group were significantly decreased compared with the normal group. Overexpression of miR-33-5p reduced the expression of JUN and FOS after MG infection (**Figures 5A, C**), while transfection with miR-33-5p inhibitor resulted in an opposite result (**Figures 5B, D**). These results show that miR-33-5p inhibits the JNK signaling pathway.

To further confirm that miR-33-5p mediates the JNK signaling pathway by negatively regulating JNK1, we assessed the effects of JNK1, JNK1 inhibitor (SP600125) and JNK1 over-expressing pcDNA3.1 vector. First, we confirmed that overexpression of JNK1 substantially increased mRNA and protein expression of JNK1, but SP600125 significantly inhibited JNK1 expression in DF-1 cells (**Figures 5E, F**). The results of Western blot and qPCR showed that overexpression of JNK1 up-regulated the expressions of JUN and FOS to activate the JNK pathway in both MG-infected and uninfected DF-1 cells (**Figures 5E, G**). Consistently, overexpression of miR-33-5p significantly reversed the elevated expression levels of p-JUN and p-FOS caused by overexpression of JNK1, whereas knockdown of JNK1 resulted in opposite results (**Figures 5F, H**).

### miR-33-5p Inhibits MG-Induced Inflammation and Apoptosis, and Reduces pMGA1.2 Expression Through JNK1

**Figure 6:**
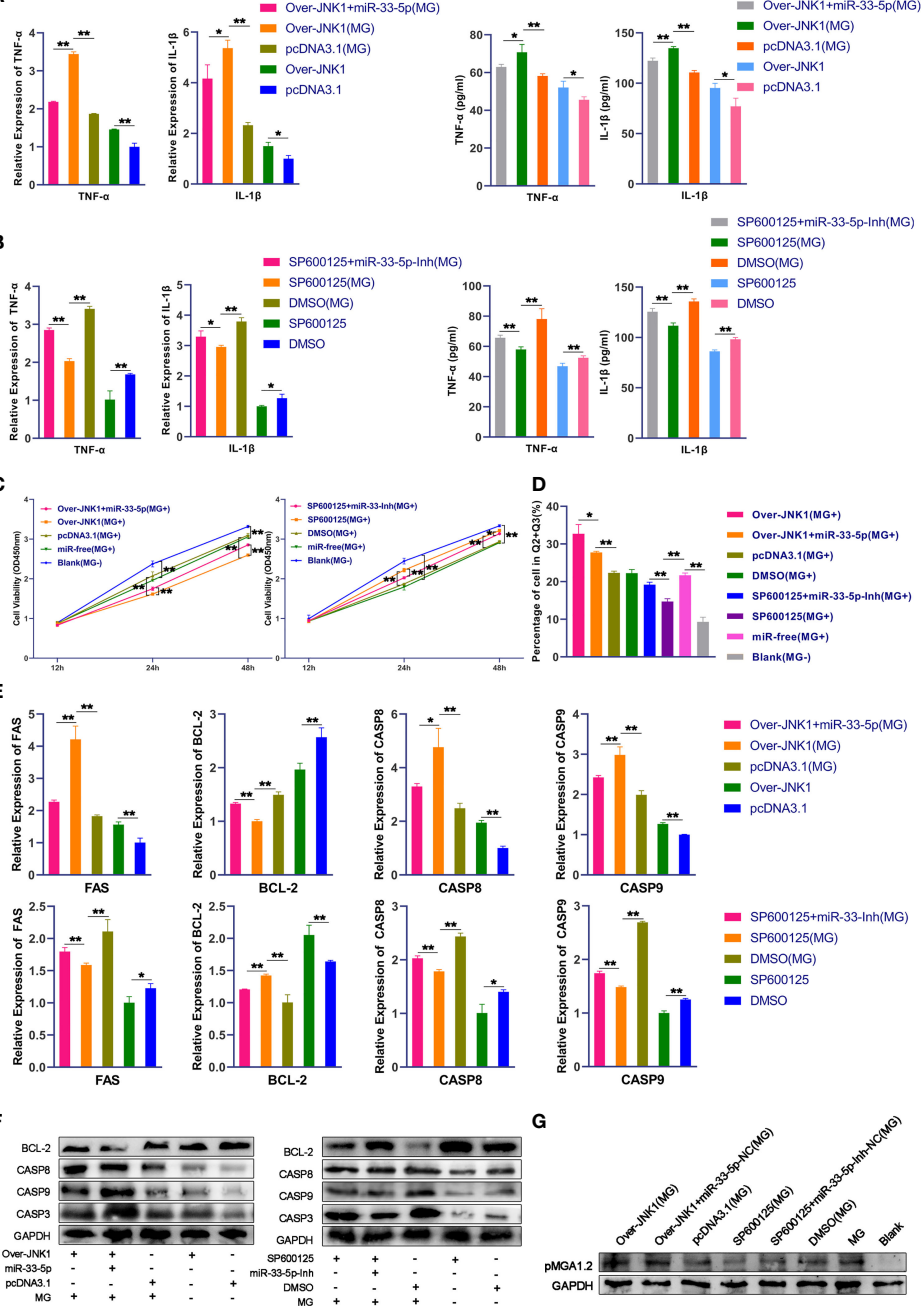
miR-33-5p inhibits MG infection-induced inflammation and apoptosis and reduces pMGA1.2 expression through JNK1. DF-1 cells were transfected with the indicated RNA oligonucleotides and infected with MG. **(A)** The expression levels of pro-inflammatory cytokines (TNF-α and IL-1β) after treatment with co-transfection of overexpression JNK1 vector and miR-33-5p mimics were detected by qPCR and Elisa; **(B)** The expression levels of pro-inflammatory cytokines (TNF-α and IL-1β) after treatment with co-transformation of SP600125 and miR-33-5p inhibitor were detected by qPCR and Elisa; **(C)** CCK-8 kit was used to detect cell proliferation at 12, 24, 48h post-infection; **(D)** The cells were stained with Annexin V- PI and analyzed by flow cytometer after 24h post-infection; **(E)** The relative mRNA expression level of apoptosis marker genes after treatment with co-transfection of overexpression JNK1 vector and miR-33-5p mimics or co-transformation of SP600125 and miR-33-5p inhibitor was detected by qPCR; **(F)** The protein expression of apoptosis marker genes treatment with co-transfection of overexpression JNK1 vector and miR-33-5p mimics or co-transformation of SP600125 and miR-33-5p inhibitor was detected by Western blot; **(G)** Western blot was used to detect the adhesion protein pMGA1.2 of MG treatment with co-transfection of overexpression JNK1 vector and miR-33-5p mimics or co-transformation of SP600125 and miR-33-5p inhibitor. GAPDH was used as the internal control. Data was expressed as the mean ± SD of three independent experiments, each with three replicates. (*p < 0.05, **p < 0.01).

To further understand the regulatory role of miR-33-5p/JNK1 on MG infection, we over-expressed or down-regulated JNK1 in MG-infected DF-1 cells through the transfection of JNK1 overexpression vector or the treatment of JNK1 inhibitor (SP600125) respectively. The results of qPCR and Western blot showed that upon MG infection, overexpression of JNK1 led to the release of pro-inflammatory factors TNF-α and IL-1β, which was opposed by the overexpression of miR-33-5p (**Figure 6A**). As expected, downregulation of JNK1 by SP600125 obviously decreased the expressions of pro-inflammatory cytokines. Interestingly, the up-regulation of TNF-α and IL-1β induced by miR-33-5p inhibitor were counteracted when co-transfected with SP600125 in MG infection groups (**Figure 6B**), indicating that miR-33-5p down-regulates the inflammation by decreasing JNK1 expression.

In addition, we examined the effect of JNK1 on cell proliferation by using the CCK-8 kit. Results showed that overexpression of JNK1 inhibited the MG-infected cell proliferation, which was partly counteracted by transfection of miR-33-5p mimics (**Figure 6C**). Then, we also examined the effect of JNK1 on cell apoptosis induced by MG infection. Flow cytometry results found that overexpression of JNK1 aggravated MG-induced apoptosis, but miR-33-5p significantly reversed this pro-apoptotic effect (**Figure 6D**). Besides, the results of Western blot and qPCR showed that JNK1 promoted the expressions of pro-apoptotic genes including FAS, Casp3, Casp8 and Casp9, but suppressed the expression of anti-apoptotic BCL-2 (**Figures 6E, F**). These results demonstrate that miR-33-5p binds to JNK1 to inhibit MG-induced cellular inflammation and apoptosis.

To further investigate the regulatory role of miR-33-5p/JNK1 on MG infection, DF-1 cells were transfected with oligonucleotides and then co-cultured with MG for 48h. As shown in **Figure 6G**, apart from the blank group, pMGA1.2 expression was significantly high in all infected groups. Compared with the control group, overexpression of JNK1 distinctly increased the pMGA1.2 expression, but knockdown of JNK1 had an inverse effect. Importantly, miR-33-5p mimics significantly reversed the elevated pMGA1.2 expression caused by JNK1 overexpression, while the miR-33-5p inhibitor attenuated the inhibitory effect of SP600125 on pMGA1.2 expression.

### Lnc90386 Is Significantly Reduced Both in MG-Infected Cells and Chicken Embryonic Lung Tissue

**Figure 7:**
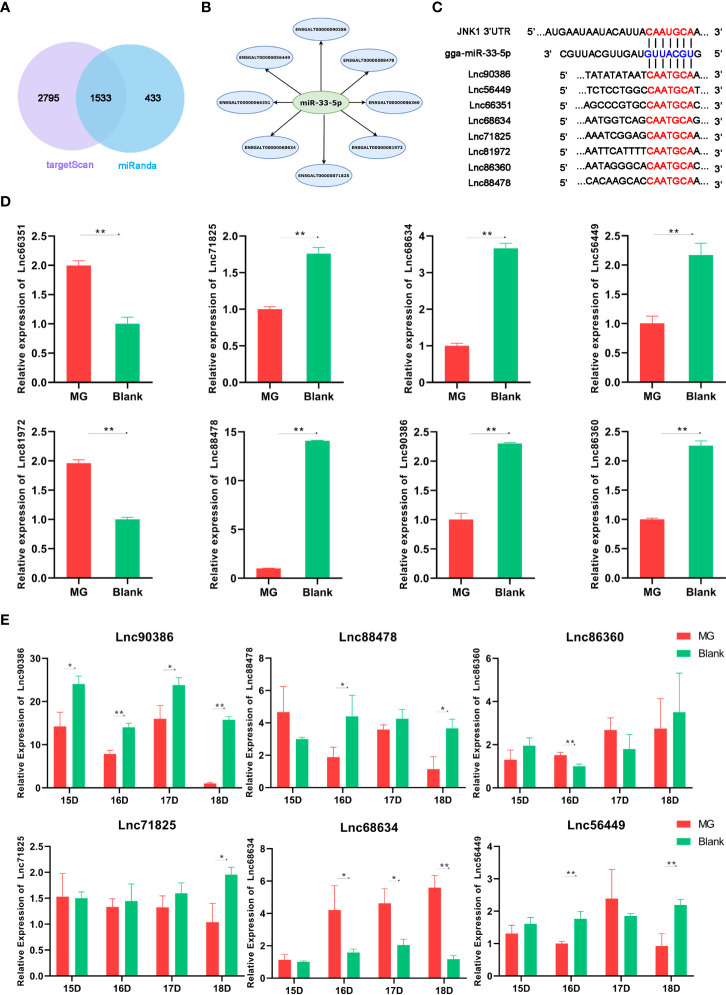
Relative lncRNA expression level in cells and chicken embryonic lung tissue. **(A)** A venn diagram depicting 1533 lncRNAs that were predicted to target miR-33-5p both by TargetScan and miRanda; **(B)** Candidate LncRNAs; **(C)** Bioinformatic database showing bindings sites of both JNK1 3’UTR and 8 lncRNAs to miR-33-5p; **(D)** DF-1 cells and CP-II cells were cultured in a 6-wells plate and treated MG. After 24h infection, we isolated the total RNA from infected cells *via* TRNzol Universal and detected the lncRNA expression by RT-qPCR; **(E)** On the 9th day of the chicken embryo hatching, MG was injected into the allantoic fluid, and the expression of lncRNA was measured by qPCR on the 15th, 16th, 17th and 18th day of the chicken embryo hatching. All data was corrected *via* GAPDH as the internal quantitative control gene. All measurements shown were the means ± SD from three independent experiments, each with three replicates. (*p < 0.05, **p < 0.01).

LncRNAs have been recently identified as ceRNAs that sponge miRNAs by complementary base pairing in animal diseases (35). To determine whether lncRNA is associated with miR-33-5p and regulates miR-33-5p expression, we obtained 14,674 chicken-related lncRNA sequences from LncRBase V.2 (http://dibresources.jcbose.ac.in/zhumur/lncrbase2/start2.php). Next, we analyzed and identified overlapping 1533 potential lncRNAs that were predicted to target miR-33-5p by both miRanda and TargetScan (**Figure 7A**). Then, eight lincRNAs were screened for candidate lncRNAs by a series of indicators including the length of lncRNAs, the score of miRanda and the localization of lncRNAs (**Figures 7B, C**). qPCR results showed that Lnc90386, Lnc56449, Lnc68634, Lnc86360, Lnc88478 and Lnc71825 were significantly down-regulated in MG-infected DF-1 cells (**Figure 7D**). Moreover, the results in MG-infected chicken embryo lung tissues showed that only Lnc90386 was extremely decreased in all MG-infected groups compared with the blank groups (**Figure 7E**). Therefore, Lnc90386 was selected for this study.

### Competitive Binding Between Lnc90386 and JNK1 for miR-33-5p

**Figure 8:**
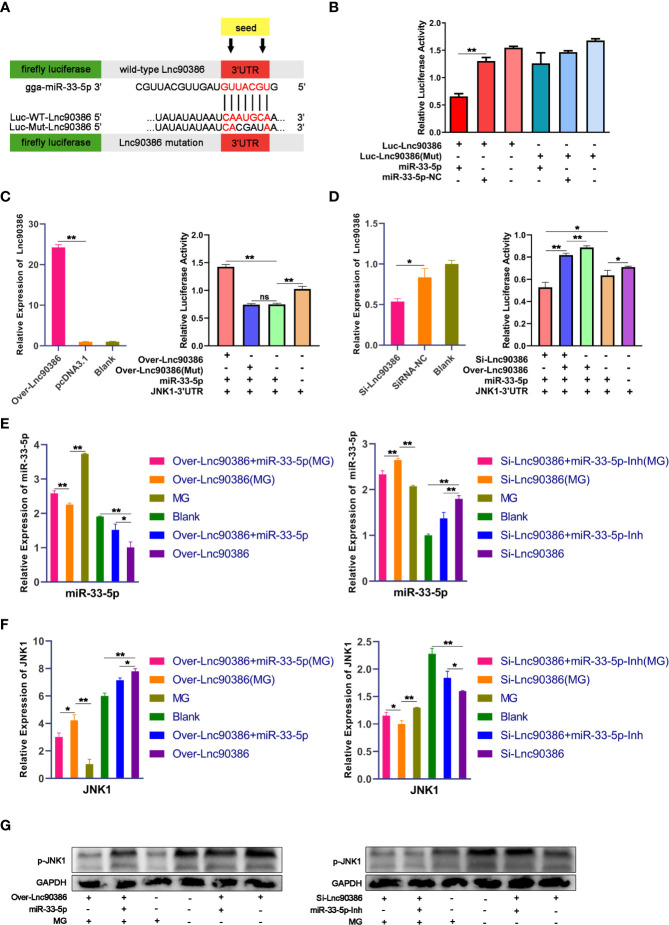
Competitive binding between Lnc90386 and JNK1 for miR-33-5p. **(A)** psiCHECK-2 dual-luciferase reporter vector containing (wild-type or mutant) Lnc90386; **(B)** DF-1 cells were co-transfected with Luc- Lnc90386 (wild-type or mutant) and the indicated RNA activity through a dual-luciferase glow assay; **(C)** DF-1 cells were co-transfected with Over-Lnc90386 (wild-type or mutant), Luc-JNK1 3′UTR and the indicated RNA. At 24h post-transfection. The expression levels of Lnc90386 after treatment with Over- Lnc90386 were detected by qPCR. The cells were assayed for both firefly and renilla activity through a dual-luciferase glow assay; **(D)** DF-1 cells were co-transfected with Si-Lnc90386, Luc-JNK1 3′UTR and the indicated RNA. At 24h post-transfection. The expression levels of Lnc90386 after treatment with Si-Lnc90386 were detected by qPCR. The cells were assayed for both firefly and renilla activity through a dual-luciferase glow assay; **(E)** The mRNA of miR-33-5p after treatment with Over- Lnc90386/Si-Lnc90386. 5s-RNA were used as the internal control; **(F)** The mRNA of JNK1 after treatment with Over- Lnc90386/Si-Lnc90386; **(G)** The protein expression of JNK1 after treatment with Over- Lnc90386/Si-Lnc90386. GAPDH was used as the internal control. (*p < 0.05, **p < 0.01).

To investigate whether Lnc90386 could function as a ceRNA in MG infection, we first constructed a luciferase construct of wild-type Lnc90386 (Luc-Lnc90386) and a mutated form [Luc-Lnc90386(Mut)] to verify the binding relationship between miR-33-5p and Lnc90386 (**Figure 8A**). Results showed that miR-33-5p mimics significantly reduced the luciferase activity of Luc-Lnc90386, yet had no effect on mutant Luc-Lnc90386(Mut) (**Figure 8B**). These results reveal that Lnc90386 might interact with miR-33-5p by this putative binding site.

Subsequently, to further verify the relationship between Lnc90386 and miR-33-5p-JNK1, wild-type/mutant vectors overexpressing Lnc90386 [named Over-Lnc90386/Over-Lnc90386(Mut)] were successfully constructed. The Lnc90386 overexpression vector had an excellent overexpression effect (**Figure 8C**). Over-Lnc90386 or Over-Lnc90386(Mut) was co-transfected with miR-33-5p mimics and JNK1-3’UTR into DF-1 cells for 24h, respectively. The results showed that the activity of JNK1-3’UTR was significantly increased when Lnc90386 was overexpressed. However, upon mutation of the miR-33-5p and Lnc90386 binding site, the fluorescence activity of JNK1-3’UTR was significantly reduced to the point where there was no statistical difference with the miR-33-5p/JNK1-3’UTR co-transfection group (**Figure 8C**). Meanwhile, the Lnc90386 knockdown (Si-Lnc90386) experiment was also performed. Si-Lnc90386 significantly decreased the relative fluorescence activity of JNK1-3’UTR. However, Over-Lnc90386 counteracted the reduced relative fluorescence activity of JNK1-3’UTR by Si-Lnc90386 (**Figure 8D**). Thus, these results show that the presence of Lnc90386 hinders the inhibitory effect of miR-33-5p on JNK1-3’UTR.

To demonstrate that Lnc90386 can act as a ceRNA to regulate JNK1 expression by competitively binding miR-33-5p. miR-33-5p mimics, miR-33-5p inhibitor, miR-33-5p mimics + Over-Lnc90386, miR-33-5p inhibitor + Si-Lnc90386 were transfected into DF-1 cells, respectively. Subsequently, DF-1 cells were infected with 200 µL of MG at 10^10^ CCU/mL at 24h post-transfection. As expected, overexpression of Lnc90386 significantly repressed the expression of miR-33-5p, but this inhibitory effect could be partially counteracted by the co-transfection of miR-33-5p. In contrast, the Si-Lnc90386 group significantly increased the expression of miR-33-5p, while the Si-Lnc90386+miR-33-5p inhibitor group significantly down-regulated the miR-33-5p expression (**Figure 8E**). Meanwhile, we also tested the JNK1 expression under the same treatment by qPCR and Western blot. As shown in **Figures 8F, G**, overexpression of Lnc90386 significantly enhanced the JNK1 expression, while knockdown of Lnc90386 had the opposite effect. When cells were co-transfected with miR-33-5p mimics and Lnc90386, miR-33-5p mimics reduced the effect of over-Lnc90386, but knockdown of Lnc90386 had the opposite effect (**Figures 8F, G**). Interestingly, compared with all uninfected groups, miR-33-5p was significantly up-regulated while JNK1 was down-regulated in all infected groups, which indicated that down-regulated Lnc90386 could promote the miR-33-5p expression and inhibit the JNK1 expression during the MG infection. The above results indicate that Lnc90386 regulates JNK1 expression by sponging miR-33-5p.

### Lnc90386 Regulates MG Infection Through miR-33-5p/JNK1

**Figure 9:**
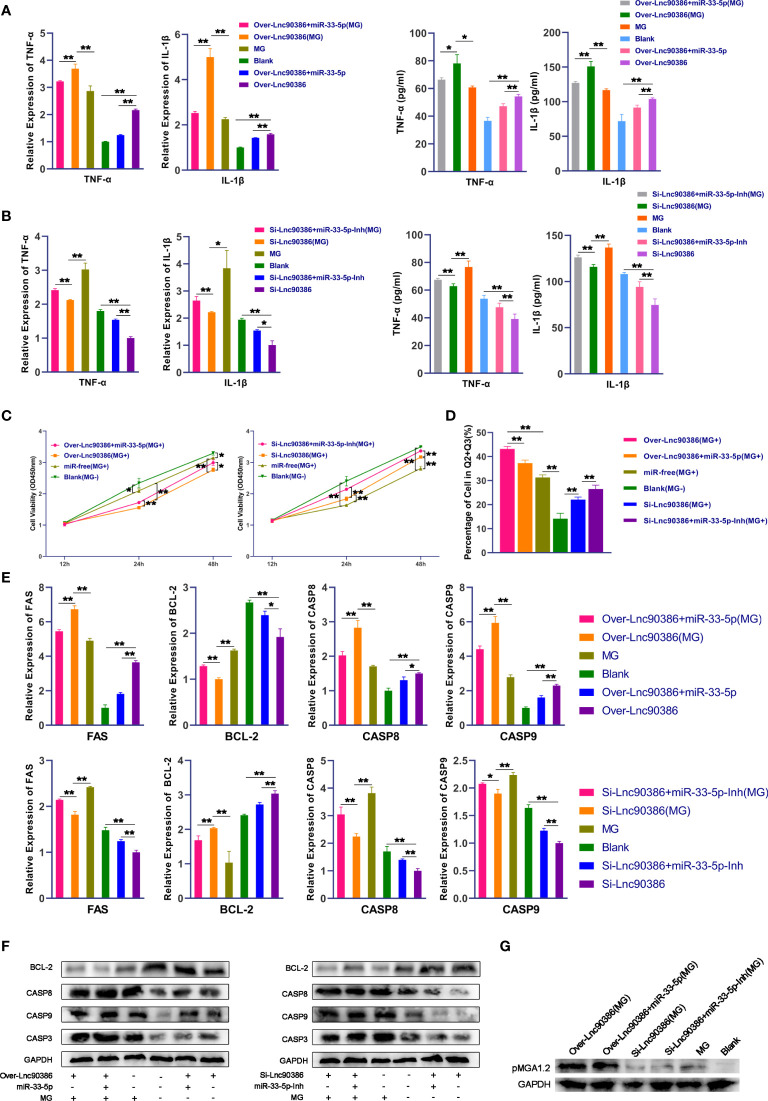
Lnc90386 regulates MG infection through miR-33-5p/JNK1. DF-1 cells were transfected with oligonucleotides and infected with MG. **(A)**The expression levels of pro-inflammatory cytokines (TNF-α and IL-1β) after treatment with co-transfection of overexpression Lnc90386 vector and miR-33-5p mimics were detected by qPCR and Elisa; **(B)** The expression levels of pro-inflammatory cytokines (TNF-α and IL-1β) after treatment with co-transformation of Si-Lnc90386 and miR-33-5p inhibitor were detected by qPCR and Elisa; **(C)** CCK-8 kit was used to detect cell proliferation at 12, 24, 48h post-infection; **(D)** The cells were stained with Annexin V- PI and analyzed by flow cytometer after 24h post-infection; **(E)** The relative mRNA expression level of apoptosis marker genes after treatment with co-transfection of overexpression Lnc90386 vector and miR-33-5p mimics or co-transformation of Si-Lnc90386 and miR-33-5p inhibitor was detected by qPCR; **(F)** The protein expression of apoptosis marker genes treatment with co-transfection of overexpression Lnc90386 vector and miR-33-5p mimics or co-transformation of Si-Lnc90386 and miR-33-5p inhibitor was detected by Western blot and qPCR; **(G)** Western blot was used to detect the adhesion protein pMGA1.2 of MG treatment with co-transfection of overexpression Lnc90386 vector and miR-33-5p mimics or co-transformation of Si-Lnc90386 and miR-33-5p inhibitor. GAPDH was used as the internal control. Data was expressed as the mean ± SD of three independent experiments, each with three replicates. (*p < 0.05, **p < 0.01).

As Lnc90386 can interact with miR-33-5p, we then tested whether Lnc90386 was capable of regulating MG infection. DF-1 cells were transfected with Over-Lnc90386, Si-Lnc90386 or co-transfected with Lnc90386 plasmid and miR-33-5p oligonucleotide, and MG was infected after 24h of the treatment. The results of qPCR and ELISA showed that overexpression of Lnc90386 resulted in up-regulation of IL-1β and TNF-α (**Figure 9A**). Interestingly, after MG infection, the up-regulation of pro-inflammatory factors induced by overexpression of Lnc90386 was partially offset by overexpression of miR-33-5p (**Figure 9A**). In contrast, knockdown of Lnc90386 down-regulated the expressions of MG-induced pro-inflammatory factors, but this trend was significantly attenuated by the miR-33-5p inhibitor (**Figure 9B**). These results indicate that Lnc90386 affects the secretion of pro-inflammatory factors by adsorbing miR-33-5p.

Subsequently, we further analyzed the effect of Lnc90386 on cell proliferation and apoptosis. As shown in **Figure 9C**, overexpression of Lnc90386 aggravated the loss of the MG-induced cell viability, which was attenuated by miR-33-5p mimic. Consistently, knockdown of Lnc90386 had an opposite effect (**Figure 9C**). Furthermore, enforced expression of Lnc90386 aggravated MG-induced apoptosis (**Figure 9D**), increased the expression of pro-apoptotic genes including FAS, Casp3, Casp8 and Casp9, and decreased the expression of the anti-apoptotic gene BCL-2 (**Figures 9E, F**). As expected, Lnc90386 siRNA had an exactly opposite effect (**Figures 9D–F**). It is worth mentioning that the miR-33-5p mimics partially reversed Lnc90386-induced cell apoptosis. Similarly, the miR-33-5p inhibitor attenuated the Lnc90386 siRNA-induced cell apoptosis inhibition.

More importantly, overexpressed Lnc90386 facilitated the expression of the MG adhesion protein pMGA1.2, while the opposite result was obtained when Lnc90386 was suppressed (**Figure 9G**). As expected, miR-33-5p inhibited the promotion effect of Lnc90386 on pMGA1.2.

## Discussion

MG infection mainly causes CRD in chicken, inducing serious inflammatory reactions and immune responses through colonization of the respiratory mucosa (36, 37). Inflammatory damage and apoptosis are MG-infected important pathogenic mechanisms. Accumulating evidence suggests that miRNAs play important regulatory roles during MG-HS infection (15). Furthermore, in recent years, lncRNAs, especially lncRNAs as ceRNAs, have been extensively documented in multiple studies including poultry diseases and immune responses (38–40). However, detailed studies regarding roles of lncRNAs and their regulatory mechanism in MG infection are still insufficient. Therefore, it would be valuable to explore the functions and underlying mechanisms of the lncRNA-miRNA-mRNA axis in MG-infected chickens.

miR-33 has been reported to regulate inflammatory responses, cellular function and gene expression (41, 42). Importantly, dysregulation of miR-33 has been frequently reported to be closely associated with respiratory disease and pathogenic microbial infections (43, 44). For example, miR-33 suppresses LPS-stimulated production of pro-inflammatory factor (TNF-α and IL-1β) in macrophages by inhibiting RIP140 expression (45). miR-33-5p regulates Angpt2-inducing mesangial cell apoptosis *via* targeting SOCS5 (46). And miR-33 inhibitor can inhibit the replication of vesicular stomatitis virus (VSV) and potentiate antiviral immune response through interacting with target gene AMPK (47). Moreover, miR-33a is negatively associated with exacerbation severity in asthmatic children, indicating their potential prognostic power for asthma management (48). Previous studies found that miR-33-5p was significantly up-regulated in MG infection by RNA-Seq (21, 31). This study showed that miR-33-5p was remarkably up-regulated both in MG-infected cells and chicken embryos. Here, we confirmed that miR-33-5p was up-regulated both in MG-infected chicken embryo lung tissue and cells, and overexpression of miR-33-5p suppressed the expression of pMGA1.2 protein, while its inhibitor resulted in the opposite result. In addition, we found that miR-33-5p inhibited MG-induced inflammation and apoptosis.

Typically, miRNAs exert regulatory effects on the gene expression and cellular processes by negatively regulating target genes at the post-transcriptional level (49). In addition, in our previous RNA-Seq study, differential miRNAs were enriched to the MAPK pathway and apoptosis pathway. It is predicted that MAPK pathway and apoptosis pathway may be the key pathways of miRNAs involved in the regulation of MG infection (21). These two pathways are consistent with this study. Meanwhile, our results also confirmed that JNK1 was a direct target of miR-33-5p. JNK1 is a member of the MAPK family. A major target of JNK1 is the transcription factor AP1 (activator protein-1, AP-1) which consists of a JNU family member and a FOS family member (50). External stimulus leads to the activation of JNK1, and then the activated p-JNK1 translocates from the cytoplasm to the nucleus and rapidly phosphorylates c-Jun to activate the activity of AP-1 (51). Activated AP-1 initiates transcription, promotes gene expression and protein synthesis, and exerts corresponding biological effects (52). In our study, we found that phosphorylated JUN and FOS were significantly up-regulated through overexpression of JNK1 and these results were consistent with the miR-33-5p inhibitor. More interestingly, overexpression of miR-33-5p could significantly reverse the increased levels of p-JNU and p-FOS induced by the overexpression of JNK1 compared with the over-JNK1 group. The above results were similar to the previous theoretical and experimental studies. Thus, it is rational to prove the role of miR-33-5p in MG infection by inhibiting JNK signaling pathway *via* directly inhibiting JNK1.

The JNK signaling pathway is known to play a role in cellular stress responses (53) and apoptosis (54), and is recently shown to modulate lung remodeling following injury (55). For instance, the activated LPS-TLR4 complex promotes the activation of JNK1 into p-JNK1, which regulates the activity of AP-1. Activated AP-1 initiates the transcription of inflammatory factors, causing the uncontrolled expression of factors such as TNF-α, ICAM-1 and nitric oxide (56). It is reported that ROS activates JNK kinase and activated JNK subsequently phosphorylates its substrate c-Jun, and then phosphorylated c-Jun further induces the activation of the caspase-3 protein, leading to apoptosis (57). Consistently, activated JNK also reduces BCL-2 expression (58). Importantly, JNK1 plays an important role in the occurrence and development of pulmonary fibrosis by affecting the phosphorylation level of JNK and lung fibroblasts proliferation. Therefore, inhibiting JNK1 will become a new strategy for anti-asthmatic airway remodeling (59). Moreover, knockdown of JNK reduces the levels of TNF-α, IL6 and IL8, and inhibits apoptosis during NDV infection. Notably, inhibition of JNK significantly suppresses NDV virus proliferation (60). These studies are similar to our findings. Specifically, we found that knockdown of JNK1 had reduced MG-induced inflammatory responses and apoptosis, and also decreased the level of pMGA1.2. In addition, miR-33-5p mimics could significantly reverse the various responses caused by overexpression of JNK1. Collectively, it is reasonable to conclude that miR-33-5p resists the MG infection through negatively regulating the JNK1/JUN/FOS pathway.

Recent studies suggest that lncRNAs are highly diverse in regulating gene expression. lncRNAs, possibly acting as endogenous sponges or ceRNAs, interact with miRNAs and influence the expression of target genes. For instance, LINC01939 directly binds to miR-17-5p and functions as a sponge of miR-17-5p to up-regulate the expression of EGR2 (the target of miR-17-5p) protein in gastric cancer (61). lncRNA LHFPL3-AS1-long directly interacts with miR-181a-5p to inhibit the mRNA degradation of BCL-2 (the target of miR-181) in melanoma stem cells (62). In addition, lncRNA HOTAIR sponges miR-326, leading to the accumulation of the miR-326 target PLAF2 in cutaneous squamous cell carcinoma (63). Similar to the above reports, in this study, we confirmed that Lnc90386 was significantly decreased in both MG-infected chicken embryos and DF-1 cells. And Lnc90386 directly bound to miR-33-5p and the presence of Lnc90386 hindered the inhibitory effect of miR-33-5p on JNK1-3’UTR. However, knockdown of Lnc90386 stimulated the expression of miR-33-5p and consequently decreased the expression of JNK1. Furthermore, lncRNAs have been systematically studied as important regulators of cell apoptosis, inflammation and pathogenic microbial infection *via* their ceRNAs function. In the model of LPS-induced lung injury, lncRNA SNHG16 is highly expressed, effectively binds to miR-146a-5p and restores CCL 5 expression, thereby promoting the inflammatory response and apoptosis (64). Wang et al. observed that lncRNA NORAD inhibits the proliferation, inflammation and fibrosis of human mesangial cells under high-glucose conditions by acting as a molecular sponge of miR-485 to modulate NRF1 expression (65). And Chu et al. found that lncRNA MARL functions as a ceRNA for miR-122 to control protein abundance of MAVS, thereby inhibiting the SCRV replication and promoting antiviral responses (66). Taken together, these findings promote us to investigate the role of Lnc90386 during MG infection. Our study found that knockdown of Lnc90386 could counteract MG infection by regulating miR-33-5p to promote cell proliferation and inhibit MG-induced inflammation and apoptosis. Collectively, Lnc90386 exerts its function by regulating miR-33-5p/JNK1 expression, implying an important role for a lncRNA–miRNA–mRNA functional network in MG infection and showing a new light on the understanding of the complex molecular mechanisms of MG-induced CRD.

## Conclusion

**Figure 10:**
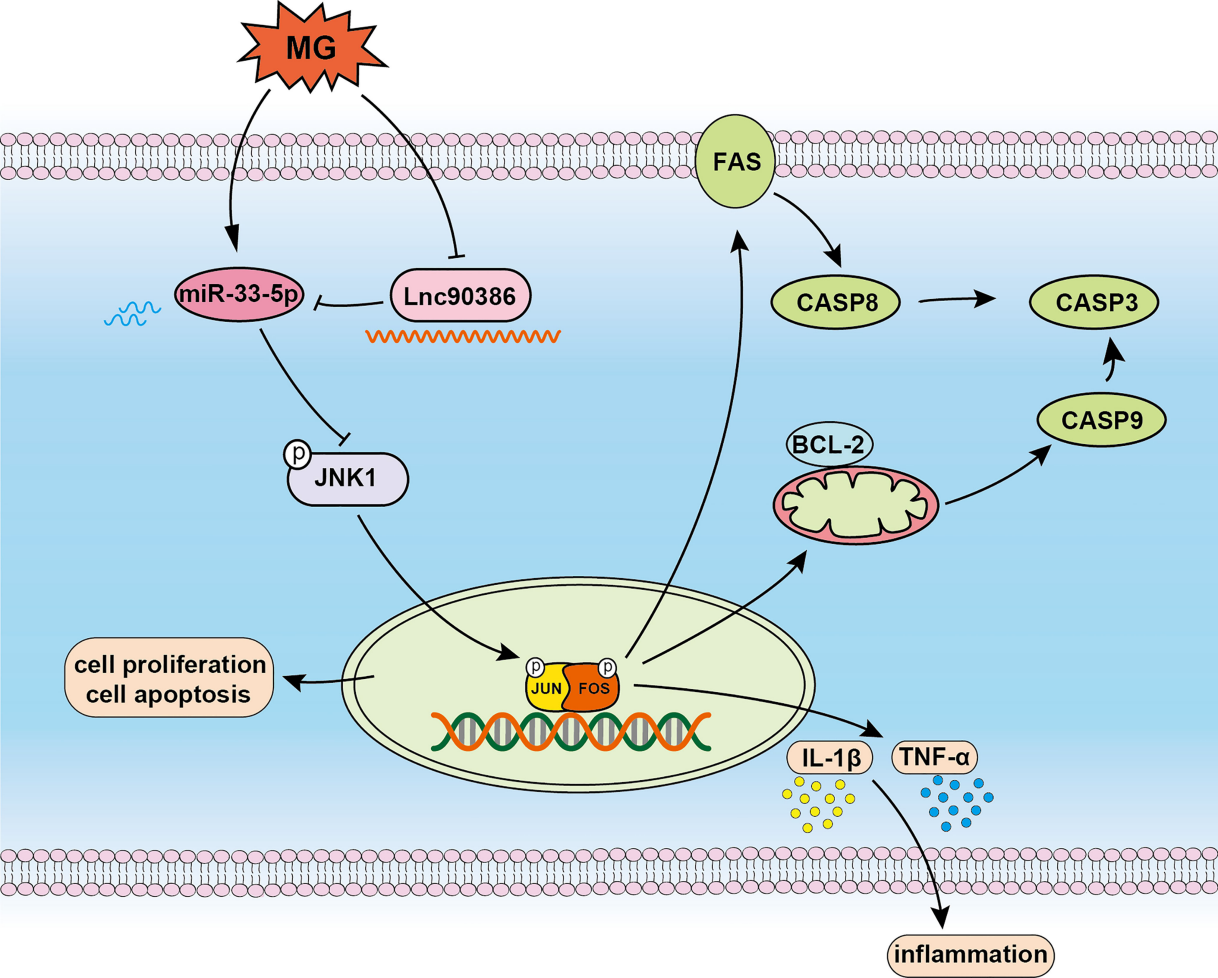
Schematic diagram of the potential roles of Lnc90386/miR-33-5p in DF-1 cells after MG infection.

In conclusion, as shown in **Figure 10**, we confirm that miR-33-5p is an anti-MG infection miRNA through targeting JNK1. Upon MG infection, down-regulated Lnc90386 acts as a ceRNA and enhances miR-33-5p expression. In MG-infected DF-1 cells, down-regulated Lnc90386 released miR-33-5p to enhance the inhibition of JNK1 by miR-33-5p, promoted cell proliferation and attenuated MG-induced inflammatory response and apoptosis to resist MG infection. Our results provide new insights into the pathogenesis of MG-induced CRD. It is possible that the regulation of miR-33-5p and Lnc90386 may provide an interesting pathway for anti-MG infection.

## Data availability Statement

The original contributions presented in the study are included in the article/supplementary material. Further inquiries can be directed to the corresponding author.

## Ethics Statement

The animal study protocol was approved by the Ethics Committee of Huazhong Agricultural University (HZAUCH-2020-0003).

## Author Contributions

YS performed experiments, wrote the manuscript and analyzed the data. YW and MZ helped to write and revise the manuscript. TW and LW provided advice for the study. XP conceived and designed the study and helped to revise the discussion. All authors contributed to the article and approved the submitted version.

## Funding

This study was supported by the National Natural Science Foundation of China (Grant No. 31972681), the National Key Research and Development Program of China (2017YFD0501500).

## Conflict of Interest

The authors declare that the research was conducted in the absence of any commercial or financial relationships that could be construed as a potential conflict of interest.

## Publisher’s Note

All claims expressed in this article are solely those of the authors and do not necessarily represent those of their affiliated organizations, or those of the publisher, the editors and the reviewers. Any product that may be evaluated in this article, or claim that may be made by its manufacturer, is not guaranteed or endorsed by the publisher.
